# Molecular and Cellular Events Mediating Glomerular Podocyte Dysfunction and Depletion in Diabetes Mellitus

**DOI:** 10.3389/fendo.2014.00151

**Published:** 2014-09-25

**Authors:** P. Anil Kumar, Gavin I. Welsh, Moin A. Saleem, Ram K. Menon

**Affiliations:** ^1^Department of Biochemistry, University of Hyderabad, Hyderabad, India; ^2^Academic Renal Unit, School of Clinical Sciences, University of Bristol, Bristol, UK; ^3^Pediatric Endocrinology and Molecular and Integrative Physiology, University of Michigan, Ann Arbor, MI, USA

**Keywords:** podocytes, growth hormone, diabetes complications, epithelial–mesenchymal transition, apoptosis

## Abstract

The essential function of the kidney is to ensure formation of a relatively protein-free ultra-filtrate, urine. The rate of filtration and composition of the primary renal filtrate is determined by the transport of fluid and solutes across the glomerular filtration barrier consisting of endothelial cells, the glomerular basement membrane, and podocyte foot processes. In diabetes mellitus (DM), components of the kidney that enable renal filtration get structurally altered and functionally compromised resulting in proteinuria that often progresses to end-stage renal disease. Histological alterations in DM include early hypertrophy of glomerular and tubular components, subsequent thickening of basement membrane in glomeruli and tubules, progressive accumulation of extracellular matrix proteins in the glomerular mesangium and loss of podocytes, together constituting a clinical condition referred to as diabetic nephropathy (DN). The glomerulus has become the focus of research investigating the mechanism of proteinuria. In particular, the progressive dysfunction and/or loss of podocytes that is contemporaneous with proteinuria in DN have attracted intense scientific attention. The absolute number of podocytes predicts glomerular function and podocyte injury is a hallmark of various glomerular diseases. This review discusses the importance of podocytes in normal renal filtration and details the molecular and cellular events that lead to podocyte dysfunction and decreased podocyte count in DN.

## Introduction

The kidneys regulate electrolyte, water, and acid–base balance and are thus indispensible for the maintenance of body homeostasis. These functions are carried out by the collective action of ~1 million nephrons in each kidney. Each nephron consists of a glomerulus and a renal tubule. The glomerulus is responsible for filtering water and small molecules from circulating plasma, while the tubular system regulates their selective reabsorption and secretion thus dictating the final composition of urine. Under normal conditions, the vertebrate kidneys ensure almost protein-free ultra-filtrated urine with tightly regulated composition. However, in disease conditions, owing to an array of abnormalities in glomerular filtration, varying amounts of plasma protein get excreted in urine. Protein concentration in urine is indexed by measuring albumin levels collected for 24 h and albuminuria is a well-known predictor of adverse renal outcome. As per the American Diabetic Association guidelines, microalbuminuria is defined as levels of albumin ranging from 30 to 300 mg in a 24-h urine collection (1). Macroalbuminuria or proteinuria is defined as a urinary albumin excretion of ≥300 mg/24 h. If left untreated, the condition of macroalbuminuria often progresses to end-stage renal disease (ESRD) warranting dialysis or renal transplant therapy. Diabetes mellitus (DM) is a group of metabolic diseases characterized by hyperglycemia resulting from defects in insulin secretion, insulin action, or both (2). Chronic hyperglycemia in DM is associated with long-term damage, dysfunction, and failure of different organs, especially eyes, kidneys, nerves, heart, and blood vessels (2). Diabetic nephropathy (DN) is a major chronic complication in diabetic subjects that develops in 20–40% of patients with Type 1 or Type 2 DM (3). Prominent early renal changes in DM include glomerular hyperfiltration, renal hypertrophy, and microalbuminuria. With advancement of the renal involvement in DM, there is a significant decrease in glomerular filtration rate (GFR) and the development of macroalbuminuria that often progresses to ESRD. In the US, DN is the most common cause of ESRD accounting for ~54% of new cases of ESRD (3).

**Figure 1:**
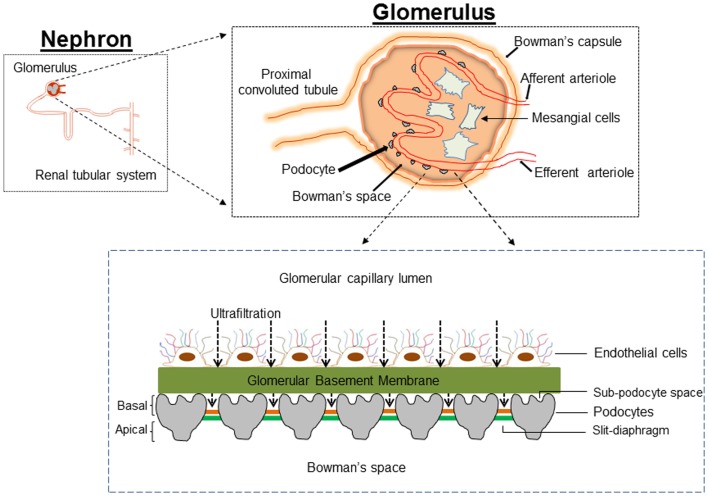
**Cartoon depicting the architecture of the glomerulus filtration barrier, which is composed of three layers: the endothelium, glomerular basement membrane, and podocytes**. Podocytes extend numerous lamellipodia that branch into primary and secondary processes, which further ramify into smaller foot processes. Foot processes from neighboring podocytes interdigitate and are connected by a modified adherent junction called slit-diaphragm (SD) that provides intercellular space for the passage of glomerular filtrate.

The original histological description by Kimmelstiel and Wilson emphasized an increase in mesangial matrix (hyalinization) as the major characteristic of renal injury in DM (4). For several decades, mesangial cells were the focus of intensive research on the assumption that changes in mesangial cells could provide the cellular and molecular basis for DN. Transforming growth factor-β1 (TGF-β1) mediates proliferation and hypertrophy of mesangial cells thus contributing to the glomerular hypertrophy in DM (5, 6). Mesangial matrix expansion with accumulation of matrix in the mesangial area reduces the capillary surface area available for filtration, correlates with proteinuria, and contributes to the progressive loss of renal function (7–9). However, the genesis of proteinuria in DM is not readily explained by the associated mesangial matrix expansion. Appearance of protein in the urine and other early features in DN indicate damage to the glomerular filtration barrier (GFB). Therefore, a “mesangiocentric” dogma explaining diabetic proteinuria cannot easily explain the pathogenesis of the disease and consideration should be given to alterations of the GFB in DM. The GFB of kidney is a size and shape dependent selective molecular sieve that tightly regulates the filtration of large macromolecules while allowing passage of small molecules and water. The three components that constitute GFB are the fenestrated glomerular endothelium, the glomerular basement membrane (GBM), and the visceral epithelial cells or podocytes (Figure 1). There is much debate as to the role of each of the components of glomerulus in the pathophysiology of proteinuria in DN. Deckert et al. proposed that endothelial dysfunction (microangiopathy) is a causal factor in the pathogenesis of proteinuria (10). Collagen accumulation and thickening of GBM during DM and loss of charge selectivity in the GBM has also been proposed to partly explain the proteinuria (11, 12). However, a decrease in negatively charged proteoglycans of GBM occurs late in the course of DN, sometimes long after the appearance of microalbuminuria, suggesting a role for the other components of the GFB in the pathogenesis of proteinuria in DN (13).

The final barrier that restricts entry of plasma proteins from the circulation into the urine is the podocyte. Whereas all the three components of GFB are required for normal renal filtration, data obtained over the past decade has highlighted the crucial role of podocytes in this filtering process (14). Studies in both patients with DM and animal models of DM revealed that onset of proteinuria is associated with decreased density and altered morphology of the podocytes (15, 16). Reduction in podocyte number has been shown to predict progressive decline in renal function and proteinuria in Pima Indians with type 2 DM (17). Studies by Peterman et al. in streptozotocin-induced diabetic rats suggest that podocytes detach from GBM into urinary space (18). Furthermore, podocytes in urine are viable and can be cultured (19, 20). A key feature of the podocyte that differentiates it from other components of the GFB is lack of a proliferative mechanism in response to injury; mesangial and glomerular endothelial cells readily proliferate in response to injury caused by an array of insults (21, 22). Thus, podocytes exit the cell-cycle to remain terminally differentiated with a quiescent phenotype. It is hypothesized that with loss of a critical proportion of the podocyte population from the glomerulus, the remaining cells are unable to compensate for the glomerular filtration function and this results in glomerulosclerosis (23). Two mechanisms are proposed to explain the loss of podocytes: (i) apoptosis and (ii) detachment. Apoptosis of podocytes was proposed as a mechanism of podocyte loss and glomerulosclerosis in TGF-β1 transgenic mice, CD2AP^−/−^ mice and puromycin aminonucleoside (PAN)-treated rats (24–26) and it has been argued that ~90% of podocytes detected in urine are apoptotic (15). However, if this is the case, it is not clear how podocytes from urine could be viable and can be cultured. Alternatively, a decreased podocyte count could be explained by impaired podocyte adhesion to the GBM. Evidence for this mechanism is provided by data showing elevated expression of anti-adhesive proteins and integrin receptors in DN (18, 20, 27, 28). Since reduction in podocyte density and urinary excretion of podocytes is an early pathological feature in patients with DM and animal models of DM (29–31), podocyte depletion could be considered as a hallmark of human and experimental DN. Although, several observations identify podocyte depletion as one of the earliest cellular features of DN, the molecular pathways and pathological mechanism(s) that manifest as decreased podocyte count in DM have only been partially characterized. In this review, we discuss the importance of podocytes in normal renal filtration and summarize systemic, cellular, and molecular events that underlie the dysfunction and/or loss of podocytes in DM.

## The Function of Podocytes

Podocytes are highly branched, terminally differentiated visceral epithelial cells of the renal glomerulus that cover the urinary side of the GBM and play a crucial role in the regulation of glomerular function. Podocytes account for about 30% of all glomerular cells. The first description of podocyte was by Karl Zimmerman in 1929 (32). He described a heavily branched cell type within the renal glomerulus. The unique architecture of podocytes includes a voluminous cell body with all major organelles, major (primary) processes, and a large number of secondary processes or foot processes. The major processes are composed of microtubules and vimentin intermediate filaments, while the foot processes are made of actin filaments. The actin cytoskeleton of the foot processes plays a critical role in the attachment of podocytes to the GBM, which together constitute a contractile apparatus that counteracts the expansive forces of the vasculature (33). Foot processes of neighboring podocytes are connected with an adherent junction named a slit-diaphragm (SD), which represents the only cell–cell contact between podocytes. The SD dictates the glomerular permselectivity and is freely permeable to water and small solutes but is a size selective barrier to the passage of large molecular weight molecules. Several proteins (Nephrin, CD2-associated protein, ZO-1, podocin, P-cadherin) determine the SD structure and enable it to act as a size and shape selective glomerular barrier. The podocyte surface is divided into two parts: the apical membrane and the basal membrane, which are above and below the SD, respectively. The apical membrane of podocyte is strongly negatively charged due to the presence of the glycoprotein glycocalyxin. Glycocalyxin repels negatively charged serum albumin and keeps adjacent FPs separated from each other (34). The basal membrane of podocytes mediates its anchorage to GBM via integrins.

Alteration in the morphology of the podocytes from the disruption of foot process architecture or the loss of entire podocytes is associated with significant proteinuria in glomerular diseases including DN (35–37). In DN, podocyte number is markedly reduced (with associated podocyturia), the foot process width is significantly widened, and the SD becomes narrower as the GFR declines (17, 29, 31, 37). The space between the underside of podocyte cell body/primary processes and the foot processes, referred to as the sub-podocyte space, covers 50–65% of the filtration surface of the GFB. The sub-podocyte space contributes to both ultrafiltration and hydraulic resistance and thus plays an important role in glomerular permeability. Significant podocyte injury and dysfunction in DM may is associated with foot process retraction and flattening (known as effacement), which enhances the loss of protein into the primary urine by altering the area and architecture of the sub-podocyte space (38).

A landmark in podocyte biology was the study that discovered mutations in nephrin (*NPHS1*) as a cause of congenital nephrotic syndrome in humans, characterized by massive proteinuria *in utero* and nephrosis at birth (39). Subsequent studies localized nephrin to the SD of podocytes and suggested that nephrin also acts as a signaling molecule, controls cytoskeletal architecture, and impact the shape and viability of podocytes. Appropriate level of expression of nephrin is necessary for normal glomerular function. Thus, in addition to diseases caused by mutations in *NPHS1*, reduction in *NPHS1* expression is also closely associated with the development of albuminuria, as observed in experimental models of both diabetes and hypertension (40, 41). Mutations in a number of other genes were subsequently identified as being associated with proteinuria and podocyte abnormalities; these include podocin (*NPHS2*), α-actinin-4 (*ACTN4*), laminin β2 (*LAMB2*), transient receptor protein 6 ion channel (*TRPC6*) and phospholipase Cε1 (*PLCE1*). In addition to serving as a size selective barrier, podocytes offer back-up support for capillaries to filter efficiently and also synthesize components of GBM (type IV collagen). Both, quantity and quality of the podocyte are critical in maintaining permselectivity of the glomerular filtration. Podocyte injury is the leading causes of chronic kidney disease in patients requiring renal replacement therapy (42). Podocytes are exposed to various noxious stimuli in DM such as high glucose, fatty acids, growth factors, cytokines, and hormones. It is generally believed that podocytes are terminally differentiated non-regenerative cells with limited healing capacity. Thus, apoptosis leads to an irreversible decrease in the number of podocytes and the filtration barrier becomes incomplete, allowing blood proteins to penetrate this filtration membrane. Although podocytes are visceral epithelial cells, they also express mesenchymal markers. Podocyte transition to a more mesenchymal nature can result in their detachment and loss from GBM and consequent impairment of renal filtration.

## Direct Effect of Hyperglycemia on Podocyte Apoptosis via Increase in Oxidative Stress

One of the major consequences of DM is hyperglycemia. There is a strong case for hyperglycemia injuring podocytes and perturbing their structural integrity, viability, and normal function. Podocytes express glucose transporters (GLUT1 and 4) and treatment with insulin increased the glucose uptake predominantly via GLUT4 (43). It was shown, both *in vivo* and *in vitro*, that high glucose induces podocyte apoptosis and contributes to reduced podocyte number (44). Susztak et al., were the first to report that in podocytes elevated concentration of extracellular glucose increased reactive oxygen species (ROS) via NADPH oxidase and induced apoptosis by activation of mitogen activated protein kinase (MAPK) and the caspase-3 cascade (44). Podocyte apoptosis increases with onset of hyperglycemia in both type 1 and type 2 DM models and podocyte apoptosis coincides with the onset of albuminuria (44). Inhibition of NADPH oxidase activity with apocyanin prevented podocyte apoptosis and resultant albuminuria (44). Eid et al. further elaborated the mechanism of glucose mediated apoptosis of podocytes by demonstrating that high glucose induces ROS via sequential upregulation of cytochrome p450, its metabolite 20-hydroxyeicosatetraenoic acid (20-HETE), subsequent increase in the NADPH oxidase (Nox1 and Nox4) expression, and NADPH-dependent superoxide anion generation (45). It was also shown that treatment of podocytes with 20-HETE mimicked the effect of high glucose and induced podocyte apoptosis. Inhibition of cytochrome P450A (CYP4A) in OVE26 mice (type 1 DM model) prevented oxidative stress and reduced both foot process effacement and apoptosis and significantly decreased albumin excretion (45). A mechanistic basis for high glucose-induced apoptosis of podocytes was shown by a study that demonstrates high glucose-induced ROS and activates TRPC6 resulting in intracellular Ca^2+^overload and apoptosis (46). In another study, it was reported that exposure of podocytes to high glucose increased intracellular Ca^2+^ concentration, leading to activation of calcineurin and subsequent nuclear accumulation of nuclear factor of activated T-cells (NFAT2) and Bax expression (47). It is noteworthy that inhibition of NFAT2 ameliorates podocyte injury and DN in db/db mice (48). Wang et al. showed that hyperglycemia induces Rho-associated coiled-coil-containing protein kinase1 (ROCK1) expression and regulates mitochondria fission by promoting phosphorylation and translocation of dynamin-related protein-1 (Drp1) into the mitochondria (49). Deletion of ROCK1 in diabetic mice prevented mitochondrial fission, whereas podocyte-specific cA-ROCK1 mice exhibited increased mitochondrial fission (49). Mitochondrial fission and consequent generation of mitochondrial ROS are implicated in podocyte apoptosis. Thus, an increase in podocyte ROS levels is considered a potential mediator of podocyte apoptosis in DM.

Inappropriate activation of the local renin–angiotensin-system (RAS) within the kidneys is also associated with renal injury. Hyperactivated RAS contributes to the elevated intraglomerular capillary pressure, promoting podocyte apoptosis in an angiotensin subtype 1 receptor (AT1R)-dependent fashion. Definitive evidence for a role for AT1R mediated signaling in podocytopathy comes from the transgenic AT1R-overexpressing rat, which spontaneously develops podocyte injury, proteinuria, and glomerulosclerosis. Pharmacologic inhibition of angiotensin II abrogates loss of nephrin and prevents foot process effacement. In addition, angiotensin receptor antagonists attenuated VEGF expression in diabetic rats and prevented the development of proteinuria. A direct role for hyperglycemia in the activation of RAS in podocytes and its implications for podocyte apoptosis was provided by a study by Durvasula and Shankland (50). They showed that exposure of podocytes to high glucose resulted in increases of angiotensin II levels via increased renin activity and AT1R levels (50).

**Figure 2:**
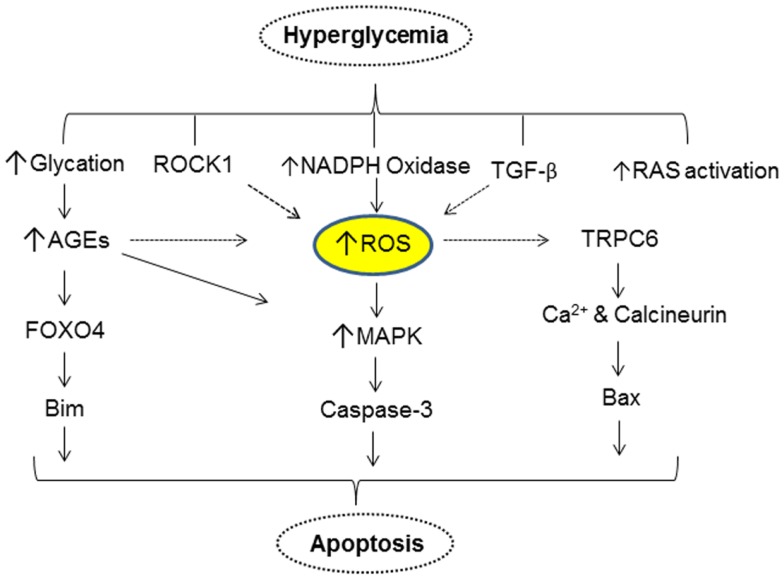
**A simplified scheme that describes hyperglycemia mediated alterations in various cellular events resulting in apoptosis of podocytes**.

One of the changes that occur as a result of elevated blood glucose in DM is the generation and accumulation of advanced glycation endproducts (AGEs). Both AGEs and their receptors (RAGE) have been shown to play a key role in the pathogenesis of DN. AGEs modified proteins activate MAP kinase and induce apoptosis. Inhibition of MAP kinase reduces the apoptotic effect of AGE modified-BSA. Exposure to AGE-BSA is associated with Akt dephosphorylation and transcriptional activation of FOXO4 leading to an increase in the expression of Bim, an effector protein of apoptosis (51). Elevated glucose levels can also result in podocyte depletion via autophagy. Thus, in a recent study it was shown that high glucose promotes autophagy of podocytes by enhanced expression of autophagic mediators LC3-2 and beclin-1 (52). Inhibition of glucose-induced autophagy by *N*-acetylcysteine argues for a role of ROS in promoting podocyte autophagy. Figure 2 provides a simplified scheme for the direct action of hyperglycemia on podocyte apoptosis.

## Role of Insulin Signaling on Podocyte Function and Apoptosis

Diabetes mellitus is characterized by reduced insulin signaling in cells resulting from insulin resistance (hallmark of type 2 DM) or lack of insulin secretion (hallmark of type 1 DM) or a combination of both mechanisms. Podocytes express the insulin receptor and are the main target of insulin action in the glomerulus, exhibiting insulin-dependent PI3K and MAPK signaling both *in vitro* and *in vivo* (43). Podocytes respond with increased glucose uptake upon exposure to insulin. The podocyte response to insulin is associated with remodeling of the actin cytoskeleton via GTPase RhoA activation and inhibition of CDC42 and this may allow for contraction of podocytes in response to increased glomerular pressure and filtration (43). Since, insulin has a critical role in normal podocyte function, it is expected that insulin-dependent cellular activities are altered in podocytes in DM. In the animal models of streptozotocin-induced insulinopenic DM and type 2 DM, glomerular insulin signaling is lost early in the progression of diabetic kidney disease (53). In non-diabetic humans, insulin resistance *per se* is associated with proteinuria. In the setting of DM, insulin resistance predicts the incidence of nephropathy in type 1 and type 2 DM (54). Podocyte-specific deletion of insulin receptor causes several glomerular abnormalities including albuminuria, increased glomerular matrix accumulation, thickening of GBM, loss of podocyte morphology, and apoptosis (55). It is noteworthy that all the above manifestations occurred under normoglycemic conditions. It was also shown that chronic exposure of podocytes to high glucose leads to decreased insulin responsiveness via increasing Src homology two domain containing phosphatase1 (SHP1) that binds to the insulin receptor and prevents downstream signaling (56). SHP1 associates with insulin receptor β to dampen insulin-stimulated Akt and extracellular signal-regulated kinase ERK phosphorylation. Analogously, in type 1 diabetic Akita mice, Akt and ERK phosphorylation were reduced in renal podocytes and this renal insulin resistance was associated with elevated SHP1 expression. The renal insulin resistance in Akita mice was associated with foot process effacement and podocyte apoptosis compared with control littermate mice. Overexpression of dominant-negative SHP1 in podocytes prevented deleterious effects of high glucose and restored insulin sensitivity (57). Impaired insulin signaling either by altered levels of fatty acids in DM and related metabolic syndrome or pro-inflammatory cytokines (IL6 and TNF-α) that accumulate during insulin resistance is implicated in podocyte injury (58). Alternatively, it was shown that ubiquitination and consequent degradation of insulin receptor substrate-1 is enhanced in hyperglycemic conditions, which manifests as impaired insulin signaling (53). Podocytes from db/db mice failed to respond to insulin treatment as evidenced by decreased AKT phosphorylation and susceptibility to cell death (59). Hence, resistance to insulin and susceptibility to cell death may partially account for the decreased podocyte number seen in early DN. The podocyte protective properties of thiazolidinediones (60) also support the argument that restoring insulin sensitivity in podocytes can ameliorate the podocytopenia observed in DM (61).

## The Role of mTOR in Podocyte Apoptosis

The mammalian target of rapamycin (mTOR) pathway is important for cellular sensing of nutrient and growth factors and cellular stress. mTOR nucleates at least two distinct multi-protein complexes, mTOR complex 1 (mTORC1) and mTOR complex 2 (mTORC2). The precise role of mTOR in the podocyte remains controversial. Activation of mTORC1 activity in podocytes resulted in proteinuria, loss of podocytes and changes in the components of GBM; all these alterations were modulated by treatment with rapamycin, an inhibitor of mTOR (54). In contrast, inhibition of mTORC1 with rapamycin treatment manifests as increased proteinuria and glomerulosclerosis in patients and animal models (54). A strong evidence for the role of mTOR in podocyte biology is provided by a study from Godel et al. (62). At 4 weeks of age, mTORC1 knockout mice developed significant albuminuria. Increased lethality was noticed at 8 months of age and was attributed to proteinuria and associated weight loss. Genetic deletion of mTORC1 in mouse podocytes induced proteinuria and progressive glomerulosclerosis. Ultrastructural analyses revealed progressive podocyte foot process broadening and effacement in mTORC1 knockout mice and indicating that mTORC1 is required to maintain podocyte function and glomerular architecture. The mTORC1 loss-of-function phenotype was reported to be similar to the phenotype observed in podocyte-specific insulin receptor deficient mice (55). Furthermore, simultaneous deletion of both mTORC1 and mTORC2 from mouse podocytes resulted in massive foot process effacement and proteinuria. These findings reveal the importance of both mTOR complexes for podocyte homeostasis. In contrast, increased mTOR activity was contemporaneous with early glomerular hypertrophy and hyperfiltration in humans with DN. Curtailing mTORC1 signaling in mice podocytes prevented glomerulosclerosis and significantly ameliorated the progression of DN. It is noteworthy that rapamycin treatment was found to be therapeutically beneficial in patients who had undergone renal replacement therapy (63, 64). In the background of DN, a more context based understanding of role of mTOR needs to be delineated. We speculate that under normal conditions, mTOR activity is essential for normal podocyte function. However, in DM elevated mTOR activity will also mediate adverse effects. Since mTOR regulates size of the cell, the role of mTOR on hypertrophy of podocytes in the early course of DN needs to be delineated. Recent studies report that mTOR hyperactivation is associated with Notch activation in podocytes, which has been shown to drive development of glomerular disease (65, 66).

## The Role of Notch Signaling in Podocyte Apoptosis

Notch constitutes an evolutionarily conserved intracellular signaling pathway that determines the cell fate. Notch pathway is activated by interaction between Notch receptors and cognate ligands from neighboring cells. Activated notch receptors are cleaved by γ-secretase and the intracellular domain translocates into the nucleus and stimulates transcription of target genes. Among the four types of Notch receptors (Notch1-4), types 1 and 2 are activated during mammalian nephrogenesis (67) and are dormant in the mature renal glomerulus (68). It was reported that Notch1 was reactivated in kidney specimens from patients with DN and focal segmental glomerulosclerosis (FSGS) and that reactivation of Notch1 correlated with the development of proteinuria due to podocyte apoptosis (65, 69). Whereas knockdown of Notch2 increased apoptosis of podocytes, a Notch2 agonistic monoclonal antibody protected injured podocytes from apoptosis via enhanced activation of AKT (70). Hence, these studies suggest that Notch signaling plays a role in pathogenesis of glomerular diseases and represents a novel therapeutic target.

## TGF-β Mediates Podocyte Apoptosis

Diabetic kidney disease is associated with increased expression of TGF-β1 in glomerular and tubular epithelial cells. TGF-β is a prototypical and multifunctional cytokine involved in many cellular processes including cell growth, differentiation, and apoptosis. The TGF-β cytokine family include TGFβ1, TGFβ2, and TGFβ3, and these ligands bind to the type II receptor, which recruits and phosphorylates the type I receptor. The type I receptor then phosphorylates receptor-regulated SMADs (Sma and Mad Related Family proteins; SMAD1, SMAD2, SMAD3, SMAD5, and SMAD8) that can bind the co-mediator SMADs (SMAD4 and SMAD10). The complex of receptor-regulated SMADs and co-mediator SMADs accumulate in the nucleus, act as transcription factors and regulate target gene expression. There are two inhibitory SMADs (SMAD6 and SMAD7) that regulate TGF-β signaling in a negative feedback manner. Several lines of evidence suggest increased levels of TGF-β in injured kidneys from experimental animals and in humans with chronic kidney diseases (71, 72). Whereas TGF-β initiates and terminates tissue repair and wound healing, sustained production of TGF-β triggers the development of tissue fibrosis, accumulation of various components of the extracellular matrix (ECM), and glomerulosclerosis (73). An in-depth discussion of the fibrogenic role of TGF-β1 is beyond the scope of this review.

Evidence for a pathogenic role for TGF-β1 in promoting podocyte apoptosis was obtained in a study by Schiffer et al. (24). Apoptosis of podocytes was observed in TGF-β1 transgenic mice and activation of p38 MAP kinase and caspase-3 was required for TGF-β1 mediated podocyte apoptosis. This study also highlighted the fact that apoptosis of podocytes occur as an early event in the course of progressive glomerulosclerosis and precedes mesangial expansion. Smad7 expression is strongly induced both in cultured podocytes treated with TGF-β and in podocytes from TGF-β1 transgenic mice (24). Unlike TGF-β1, Smad7 induces podocyte apoptosis by inhibiting nuclear translocation and transcriptional activity of NF-κB. However, co-expression of Smad7 has an additive effect on the TGF-β1 mediated podocyte apoptosis (24). Recently, an alternative theory was put forward for TGF-β1 mediated podocyte injury. TGF-β1 increases mitochondrial membrane potential and oxygen consumption rate via mTOR pathway, resulting in increased ROS generation and podocyte injury (74). TGF-β receptor-SMAD axis dependent mitochondrial Nox4 activation and ROS production impedes mitochondrial function and apoptosis (75). Several pathological mediators such as angiotensin II, VEGF, and Gremlin aggravate hyperglycemia-induced podocyte injury by a TGF-β dependent signaling pathway (76–78). Nevertheless, it is intriguing that anti-TGF-β interventions are only inconsistently associated with reduction of albuminuria in experimental models of DN (79).

## Epithelial-to-Mesenchymal Transition as a Mechanism for Podocytopenia in Diabetes Mellitus

Epithelial-to-mesenchymal transition (EMT) is an orchestrated series of events in which cell–cell and cell–ECM interactions are altered to release epithelial cells from the host tissue, the cytoskeleton is reorganized to enable these cells to migrate, and an altered transcriptional program is induced to maintain these cells in a mesenchymal phenotype (80). EMT is a fundamental process that occurs during many stages of development in which the embryonic epithelium gives rise to the mesoderm, and in delamination of the neural crest, which produces a population of highly mobile cells that migrate to and are incorporated into many different tissues (80). Nevertheless, EMT is potentially destructive if deregulated and unrestrained EMT is an integral component of the pathology of tumor metastasis and tissue fibrosis (81).

In response to injury, podocytes are capable of undergoing a phenotypic switch to attain an embryonic form by shedding their specialized epithelial characteristics and by acquiring mesenchymal features (81). It is conceivable that podocytes after undergoing EMT abandon their complex morphological architecture and relinquish their highly specialized functions, which impairs the integrity of GFB, leading to the onset of proteinuria. Although it is debatable whether EMT contributes to decreased podocyte density in diabetic kidney disease, identification of significant amount of viable urinary podocytes from both experimental models of DN and from patients with DN suggest that podocyte dropout might be caused by decreased podocyte adhesion, which is a potential consequence of EMT (20, 82). Podocytes were identified by immunostaining for nephrin and podocin in the urine from passive Heymann nephritis model of membranous nephropathy and in the streptozotocin model of DN in rats (18, 82). Podocytes isolated from urine readily adhere to tissue culture plates and are able to proliferate under standard cell culture conditions. The appearance of podocytes in urine questions the quality of adhesive proteins that maintain the integrity of podocytes with GBM. Podocytes adhere to the GBM, which is primarily comprised of collagen IV and laminins. Proteins belonging to the integrin family are crucial for cellular interactions with ECM components of GBM. α3β1 integrin is an adhesion receptor for laminins and type IV collagen, and expressed primarily on podocytes. Decreased α3β1 integrin expression was observed in podocytes exposed to high glucose concentration (83). Attenuation of α3β1 integrin expression in podocytes from both short- and long-term diabetic rats argues for a role for podocyte detachment from GBM in DN (84, 85). In a retrospective cross-sectional analysis, greater amounts of fibroblast-specific protein-1 (FSP1)-positive podocytes were observed in urinary sediments of diabetic patients with macroalbuminuria than in those with normoalbuminuria (86). FSP1-positive podocytes selectively expressed Snail1, a known trigger for EMT. This study suggests that appearance of FSP1 in podocytes of patients with DM is associated with more severe clinical and pathological findings of DN and correlates with podocyte detachment consequent to the EMT process. A list of epithelial and mesenchymal markers in podocytes is provided in Table 1.

**Table 1:** **List of EMT markers that either decrease or increase in podocytes upon treatment with GH and TGF-β1**.

Attenuated markers	Enhanced markers
E-cadherin	ZEB2
P-cadherin	Snail
Zonula Occludens-1 (ZO-1)	FSP1
Nephrin	Desmin
	α-Smooth MuscleActin
	Vimentin
	Nestin
	MMP9

## A Pathogenic Role for TGF-β1 in Podocyte EMT

In the earlier section, we discussed the role of TGF-β1 in podocyte apoptosis. Although the effect of TGF-β1 on podocyte apoptosis and thickening of GBM has been known for some time, evidence for the role of TGF-β1 in inducing phenotypic conversion of podocytes to motile mesenchymal cells was provided by study from Li et al. (87). TGF-β treatment in conditionally immortalized mouse podocytes attenuated the expression of P-cadherin, ZO-1, and nephrin, while promoting the acquisition of mesenchymal markers such as Snail, FSP1, and Desmin. As nephrin, P-cadherin, and ZO-1 are important components of SD of podocytes, decreased expression of these proteins impairs the integrity of the SD leading to foot process effacement and altered podocyte permselectivity to albumin (87). TGF-β1 induced Snail expression was implicated in initiating EMT of podocytes and ectopic expression of Snail suppressed P-cadherin and nephrin in podocytes (87). Further insights into the role of TGF-β1 in podocyte EMT was obtained by a study from Herman-Edelstein et al. (88). In this study, the authors employed immortalized human podocytes and demonstrated that treatment with TGF-β1 resulted in retraction and shortening of foot processes and contraction of the podocyte cell body. In addition to these morphological changes, exposure to TGF-β1 resulted in a dedifferentiated phenotype in podocytes with enhanced motility (88). A dose- and time-dependent attenuation of podocyte epithelial markers and acquisition of mesenchymal markers and ECM components was observed following treatment with TGF-β (88). Besides regulating the expression of EMT markers, TGF-β1 treatment decreased the expression of adhesive proteins of the podocytes. In nephrotic rats, TGF-β1 suppresses the glomerular expression of α3 integrin (89). Since the α3 integrin subunit has a pivotal role in regulating podocyte adhesiveness to GBM, it is speculated that loss of α3 subunit expression results in the detachment of podocytes from GBM. Furthermore, when cultured podocytes were treated with TGF-β1, α3β1 integrin expression was decreased with concomitant reduction in podocyte adhesion (90). To summarize, TGF-β1 exerts multiple effects on podocytes; (i) TGF-β1 reduces podocyte adhesion to the GBM via down regulation of α3β1 integrin; (ii) TGF-β1 induces EMT of podocytes by attenuating epithelial markers and acquisition of mesenchymal markers resulting in podocyte depletion; (iii) TGF-β1 impairs the architecture of SD and alters the permselectivity of podocytes, and (iv) TGF-β1 increases apoptosis of podocytes (88).

## Role of Growth Hormone in EMT and Apoptosis of Podocytes

**Figure 3:**
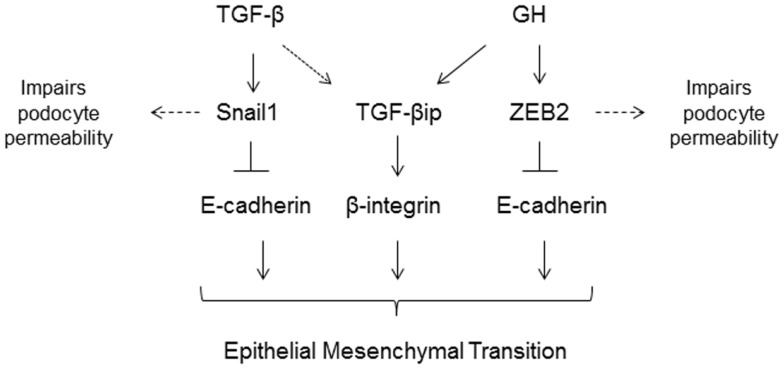
**TGF-β and growth hormone (GH) induce epithelial-to-mesenchymal (EMT) transition of podocytes via activation of Snail1 and ZEB2 genes, respectively**. Both TGF-β and GH induce expression of TGF-β induced protein (TGF-βip). In addition to inducing EMT, these transcriptional factors also suppress expression of slit-diaphragm proteins resulting in increased podocyte permeability to plasma proteins.

In type 1 DM, insulin deficiency results in impaired hepatic IGF-1 production but increased secretion of hepatic IGFBP1. Increase in IGFBP1 leads to inhibition of IGF-1 action at the cellular level and in concert with the lower levels of hepatic IGF-1 production results, via a negative feedback mechanism, in increased growth hormone (GH) secretion by the pituitary gland and higher circulatory levels of GH (91). GH excess in both humans and in transgenic animal models is characterized by significant structural and functional changes in the kidney. In humans, a direct relationship has been noted between the activity of the GH/IGF-1 axis and renal hypertrophy, macroalbuminuria, and glomerulosclerosis. Conversely, states of GH deficiency or ablation of GH receptor or its activity confer a protective effect against DN (92). A recent study revealed that podocytes express GH receptor and respond to GH by activation of JAK/STAT signaling (93). Kumar et al. demonstrated that podocytes respond to GH by inducing expression of zinc-finger E-box binding protein (ZEB2), a transcription factor that mediates EMT (94). It was also shown that GH treatment resulted in loss of E- and P-cadherin expression and consequently attenuated podocyte permselectivity to albumin (94). Abrogation of ZEB2 expression prevented GH dependent EMT changes in podocytes (94). In ongoing studies, GH administration to rats increased enhanced apoptosis and EMT, decreased podocyte count, and increased proteinuria (Kumar et al., under review). The proposed mechanisms of GH and TGF-β mediated injury to podocytes are summarized in Figure 3.

## Concluding Remarks

Our understanding of podocyte biology has increased significantly in the past decade, and we are learning more about novel mediators of podocyte injury, apoptosis, and their detachment. The molecular mechanism(s) of podocytopenia in diabetic mellitus is now better understood. A majority of cellular events in DM exert toxicity by inducing podocyte apoptosis via increase in mitochondrial and/or cytoplasmic ROS (Figure 2), suggesting that the ROS pathway could be a pharmacological target to combat podocyte apoptosis. Snail and ZEB2 are key transcriptional factors implicated in initiating EMT and are induced by TGF-β1 and GH, respectively (Figure 3) (87, 94). It is envisaged that a greater insight into podocyte biology will lead to better therapeutic options for improving the survival and quality of life of patients with diabetic nephropathy.

## Conflict of Interest Statement

The authors declare that the research was conducted in the absence of any commercial or financial relationships that could be construed as a potential conflict of interest.

## References

[1] Association AD Standards of medical care in diabetes. Diabetes Care (2005) 28(Suppl 1):S4–36 10.2337/diacare.28.suppl_1.S4 15618112

[2] AD Association. Diagnosis and classification of diabetes mellitus. Diabetes Care (2014) 37(Suppl 1):S81–90 10.2337/dc14-S081 24357215

[3] UADRA. United States Renal Data System, N.I.O.H. of ESRD. Bethesda, MD: National Institute of Diabetes, and Digestive and Kidney Diseases (2009).

[4] Kimmelstiel P Wilson C Intercapillary lesions in the glomeruli of the kidney. Am J Pathol (1936) 12:83–98 19970254 PMC1911022

[5] Wolf G Ziyadeh FN Molecular mechanisms of diabetic renal hypertrophy. Kidney Int (1999) 56:393–405 10.1046/j.1523-1755.1999.00590.x 10432377

[6] Wolf G Sharma K Chen Y Ericksen M Ziyadeh FN High glucose-induced proliferation in mesangial cells is reversed by autocrine TGF-beta. Kidney Int (1992) 42:647–56 10.1038/ki.1992.330 1357223

[7] Mauer SM Steffes MW Ellis EN Sutherland DE Brown DM Goetz FC Structural-functional relationships in diabetic nephropathy. J Clin Invest (1984) 74:1143–55 10.1172/JCI111523 6480821 PMC425280

[8] Osterby R Gall MA Schmitz A Nielsen FS Nyberg G Parving HH Glomerular structure and function in proteinuric type 2 (non-insulin-dependent) diabetic patients. Diabetologia (1993) 36:1064–70 10.1007/BF02374500 8243856

[9] White KE Bilous RW Type 2 diabetic patients with nephropathy show structural-functional relationships that are similar to type 1 disease. J Am Soc Nephrol (2000) 11:1667–73 10966491 10.1681/ASN.V1191667

[10] Deckert T Feldt-Rasmussen B Borch-Johnsen K Jensen T Kofoed-Enevoldsen A Albuminuria reflects widespread vascular damage. The steno hypothesis. Diabetologia (1989) 32:219–26 10.1007/BF00285287 2668076

[11] Kefalides NA Basement membrane research in diabetes mellitus. Coll Relat Res (1981) 1:295–9 10.1016/S0174-173X(81)80006-4 6286230

[12] Menne J Park JK Boehne M Elger M Lindschau C Kirsch T Diminished loss of proteoglycans and lack of albuminuria in protein kinase C-alpha-deficient diabetic mice. Diabetes (2004) 53:2101–9 10.2337/diabetes.53.8.2101 15277392

[13] Vernier RL Steffes MW Sisson-Ross S Mauer SM Heparan sulfate proteoglycan in the glomerular basement membrane in type 1 diabetes mellitus. Kidney Int (1992) 41:1070–80 10.1038/ki.1992.163 1513088

[14] Patrakka J Tryggvason K New insights into the role of podocytes in proteinuria. Nat Rev Nephrol (2009) 5:463–8 10.1038/nrneph.2009.108 19581907

[15] Shankland SJ The podocyte’s response to injury: role in proteinuria and glomerulosclerosis. Kidney Int (2006) 69:2131–47 10.1038/sj.ki.5000410 16688120

[16] Wolf G Chen S Ziyadeh FN From the periphery of the glomerular capillary wall toward the center of disease: podocyte injury comes of age in diabetic nephropathy. Diabetes (2005) 54:1626–34 10.2337/diabetes.54.6.1626 15919782

[17] Meyer TW Bennett PH Nelson RG Podocyte number predicts long-term urinary albumin excretion in Pima Indians with Type II diabetes and microalbuminuria. Diabetologia (1999) 42:1341–4 10.1007/s001250051447 10550418

[18] Petermann AT Krofft R Blonski M Hiromura K Vaughn M Pichler R Podocytes that detach in experimental membranous nephropathy are viable. Kidney Int (2003) 64:1222–31 10.1046/j.1523-1755.2003.00217.x 12969140

[19] Mundel P Urinary podocytes: lost and found alive. Kidney Int (2003) 64:1529–30 10.1046/j.1523-1755.2003.00339.x 12969175

[20] Vogelmann SU Nelson WJ Myers BD Lemley KV Urinary excretion of viable podocytes in health and renal disease. Am J Physiol Renal Physiol (2003) 285:F40–8 10.1152/ajprenal.00404.2002 12631553 PMC3368602

[21] Kriz W Progressive renal failure – inability of podocytes to replicate and the consequences for development of glomerulosclerosis. Nephrol Dial Transplant (1996) 11:1738–42 10.1093/oxfordjournals.ndt.a027660 8918614

[22] Shankland SJ Hugo C Coats SR Nangaku M Pichler RH Gordon KL Changes in cell-cycle protein expression during experimental mesangial proliferative glomerulonephritis. Kidney Int (1996) 50:1230–9 10.1038/ki.1996.432 8887282

[23] Fukuda A Wickman LT Venkatareddy MP Sato Y Chowdhury MA Wang SQ Angiotensin II-dependent persistent podocyte loss from destabilized glomeruli causes progression of end stage kidney disease. Kidney Int (2012) 81:40–55 10.1038/ki.2011.306 21937979 PMC3739490

[24] Schiffer M Bitzer M Roberts IS Kopp JB ten Dijke P Mundel P Apoptosis in podocytes induced by TGF-beta and Smad7. J Clin Invest (2001) 108:807–16 10.1172/JCI200112367 11560950 PMC200928

[25] Schiffer M Mundel P Shaw AS Bottinger EP A novel role for the adaptor molecule CD2-associated protein in transforming growth factor-beta-induced apoptosis. J Biol Chem (2004) 279:37004–12 10.1074/jbc.M403534200 15213232

[26] Kim YH Goyal M Kurnit D Wharram B Wiggins J Holzman L Podocyte depletion and glomerulosclerosis have a direct relationship in the PAN-treated rat. Kidney Int (2001) 60:957–68 10.1046/j.1523-1755.2001.060003957.x 11532090

[27] Pozzi A Jarad G Moeckel GW Coffa S Zhang X Gewin L Beta1 integrin expression by podocytes is required to maintain glomerular structural integrity. Dev Biol (2008) 316:288–301 10.1016/j.ydbio.2008.01.022 18328474 PMC2396524

[28] Durvasula RV Shankland SJ Podocyte injury and targeting therapy: an update. Curr Opin Nephrol Hypertens (2006) 15:1–7 10.1097/01.mnh.0000199012.79670.0b 16340659

[29] Nakamura T Ushiyama C Suzuki S Hara M Shimada N Ebihara I Urinary excretion of podocytes in patients with diabetic nephropathy. Nephrol Dial Transplant (2000) 15:1379–83 10.1093/ndt/15.9.1379 10978394

[30] Siu B Saha J Smoyer WE Sullivan KA Brosius FC III Reduction in podocyte density as a pathologic feature in early diabetic nephropathy in rodents: prevention by lipoic acid treatment. BMC Nephrol (2006) 7:6 10.1186/1471-2369-7-6 16539708 PMC1435876

[31] Pagtalunan ME Miller PL Jumping-Eagle S Nelson RG Myers BD Rennke HG Podocyte loss and progressive glomerular injury in type II diabetes. J Clin Invest (1997) 99:342–8 10.1172/JCI119163 9006003 PMC507802

[32] Zimmerman KW Uber den Bau des glomerulus der menschlichen niere. Z Mikr Anat Forsch (1929) 18:520–2

[33] Ronco P Proteinuria: is it all in the foot? J Clin Invest (2007) 117:2079–82 10.1172/JCI32966 17671644 PMC1934599

[34] Takeda T Go WY Orlando RA Farquhar MG Expression of podocalyxin inhibits cell-cell adhesion and modifies junctional properties in Madin-Darby canine kidney cells. Mol Biol Cell (2000) 11:3219–32 10.1091/mbc.11.9.3219 10982412 PMC14987

[35] Kriz W Gretz N Lemley KV Progression of glomerular diseases: is the podocyte the culprit? Kidney Int (1998) 54:687–97 10.1046/j.1523-1755.1998.00044.x 9734594

[36] Barisoni L Kriz W Mundel P D’Agati V The dysregulated podocyte phenotype: a novel concept in the pathogenesis of collapsing idiopathic focal segmental glomerulosclerosis and HIV-associated nephropathy. J Am Soc Nephrol (1999) 10:51–61 9890309 10.1681/ASN.V10151

[37] Bjorn SF Bangstad HJ Hanssen KF Nyberg G Walker JD Viberti GC Glomerular epithelial foot processes and filtration slits in IDDM patients. Diabetologia (1995) 38:1197–204 10.1007/BF00422369 8690172

[38] Salmon AH Toma I Sipos A Muston PR Harper SJ Bates DO Evidence for restriction of fluid and solute movement across the glomerular capillary wall by the subpodocyte space. Am J Physiol Renal Physiol (2007) 293:F1777–86 10.1152/ajprenal.00187.2007 17804486

[39] Kestila M Lenkkeri U Mannikko M Lamerdin J McCready P Putaala H Positionally cloned gene for a novel glomerular protein – nephrin – is mutated in congenital nephrotic syndrome. Mol Cell (1998) 1:575–82 10.1016/S1097-2765(00)80057-X 9660941

[40] Bonnet F Cooper ME Kawachi H Allen TJ Boner G Cao Z Irbesartan normalises the deficiency in glomerular nephrin expression in a model of diabetes and hypertension. Diabetologia (2001) 44:874–7 10.1007/s001250100546 11508272

[41] Forbes JM Bonnet F Russo LM Burns WC Cao Z Candido R Modulation of nephrin in the diabetic kidney: association with systemic hypertension and increasing albuminuria. J Hypertens (2002) 20:985–92 10.1097/00004872-200205000-00034 12011660

[42] Estacio RO Schrier RW Diabetic nephropathy: pathogenesis, diagnosis, and prevention of progression. Adv Intern Med (2001) 46:359–408 11147259

[43] Coward RJ Welsh GI Yang J Tasman C Lennon R Koziell A The human glomerular podocyte is a novel target for insulin action. Diabetes (2005) 54:3095–102 10.2337/diabetes.54.11.3095 16249431

[44] Susztak K Raff AC Schiffer M Bottinger EP Glucose-induced reactive oxygen species cause apoptosis of podocytes and podocyte depletion at the onset of diabetic nephropathy. Diabetes (2006) 55:225–33 10.2337/diabetes.55.01.06.db05-0894 16380497

[45] Eid AA Gorin Y Fagg BM Maalouf R Barnes JL Block K Mechanisms of podocyte injury in diabetes: role of cytochrome P450 and NADPH oxidases. Diabetes (2009) 58:1201–11 10.2337/db08-1536 19208908 PMC2671039

[46] Liu BC Song X Lu XY Li DT Eaton DC Shen BZ High glucose induces podocyte apoptosis by stimulating TRPC6 via elevation of reactive oxygen species. Biochim Biophys Acta (2013) 1833:1434–42 10.1016/j.bbamcr.2013.02.031 23499875 PMC4134943

[47] Li R Zhang L Shi W Zhang B Liang X Liu S NFAT2 mediates high glucose-induced glomerular podocyte apoptosis through increased Bax expression. Exp Cell Res (2013) 319:992–1000 10.1016/j.yexcr.2013.01.007 23340267

[48] Zhang L Li R Shi W Liang X Liu S Ye Z NFAT2 inhibitor ameliorates diabetic nephropathy and podocyte injury in db/db mice. Br J Pharmacol (2013) 170:426–39 10.1111/bph.12292 23826864 PMC3834765

[49] Wang W Wang Y Long J Wang J Haudek SB Overbeek P Mitochondrial fission triggered by hyperglycemia is mediated by ROCK1 activation in podocytes and endothelial cells. Cell Metab (2012) 15:186–200 10.1016/j.cmet.2012.01.009 22326220 PMC3278719

[50] Durvasula RV Shankland SJ Activation of a local renin angiotensin system in podocytes by glucose. Am J Physiol Renal Physiol (2008) 294:F830–9 10.1152/ajprenal.00266.2007 18216149

[51] Chuang PY Yu Q Fang W Uribarri J He JC Advanced glycation endproducts induce podocyte apoptosis by activation of the FOXO4 transcription factor. Kidney Int (2007) 72:965–76 10.1038/sj.ki.5002456 17667983 PMC3191877

[52] Ma T Zhu J Chen X Zha D Singhal PC Ding G High glucose induces autophagy in podocytes. Exp Cell Res (2013) 319:779–89 10.1016/j.yexcr.2013.01.018 23384600 PMC3628680

[53] Mima A Ohshiro Y Kitada M Matsumoto M Geraldes P Li C Glomerular-specific protein kinase C-beta-induced insulin receptor substrate-1 dysfunction and insulin resistance in rat models of diabetes and obesity. Kidney Int (2011) 79:883–96 10.1038/ki.2010.526 21228767 PMC3612886

[54] Brosius FC Coward RJ Podocytes, signaling pathways, and vascular factors in diabetic kidney disease. Adv Chronic Kidney Dis (2014) 21:304–10 10.1053/j.ackd.2014.03.011 24780459 PMC4075065

[55] Welsh GI Hale LJ Eremina V Jeansson M Maezawa Y Lennon R Insulin signaling to the glomerular podocyte is critical for normal kidney function. Cell Metab (2010) 12:329–40 10.1016/j.cmet.2010.08.015 20889126 PMC4949331

[56] Geraldes P Hiraoka-Yamamoto J Matsumoto M Clermont A Leitges M Marette A Activation of PKC-delta and SHP-1 by hyperglycemia causes vascular cell apoptosis and diabetic retinopathy. Nat Med (2009) 15:1298–306 10.1038/nm.2052 19881493 PMC3290906

[57] Drapeau N Lizotte F Denhez B Guay A Kennedy CR Geraldes P Expression of SHP-1 induced by hyperglycemia prevents insulin actions in podocytes. Am J Physiol Endocrinol Metab (2013) 304:E1188–98 10.1152/ajpendo.00560.2012 23531619

[58] Lennon R Pons D Sabin MA Wei C Shield JP Coward RJ Saturated fatty acids induce insulin resistance in human podocytes: implications for diabetic nephropathy. Nephrol Dial Transplant (2009) 24:3288–96 10.1093/ndt/gfp302 19556298 PMC7614380

[59] Tejada T Catanuto P Ijaz A Santos JV Xia X Sanchez P Failure to phosphorylate AKT in podocytes from mice with early diabetic nephropathy promotes cell death. Kidney Int (2008) 73:1385–93 10.1038/ki.2008.109 18385666

[60] Sarafidis PA Bakris GL Protection of the kidney by thiazolidinediones: an assessment from bench to bedside. Kidney Int (2006) 70:1223–33 10.1038/sj.ki.5001620 16883325

[61] Lennon R Welsh GI Singh A Satchell SC Coward RJ Tavare JM Rosiglitazone enhances glucose uptake in glomerular podocytes using the glucose transporter GLUT1. Diabetologia (2009) 52:1944–52 10.1007/s00125-009-1423-7 19533082 PMC7614273

[62] Godel M Hartleben B Herbach N Liu S Zschiedrich S Lu S Role of mTOR in podocyte function and diabetic nephropathy in humans and mice. J Clin Invest (2011) 121:2197–209 10.1172/JCI44774 21606591 PMC3104746

[63] Gamboa O Montero C Mesa L Benavides C Reino A Torres RE Cost-effectiveness analysis of the early conversion of tacrolimus to mammalian target of rapamycin inhibitors in patients with renal transplantation. Transplant Proc (2011) 43:3367–76 10.1016/j.transproceed.2011.09.092 22099798

[64] Stallone G Infante B Grandaliano G Bristogiannis C Macarini L Mezzopane D Rapamycin for treatment of type I autosomal dominant polycystic kidney disease (RAPYD-study): a randomized, controlled study. Nephrol Dial Transplant (2012) 27:3560–7 10.1093/ndt/gfs264 22785114

[65] Niranjan T Bielesz B Gruenwald A Ponda MP Kopp JB Thomas DB The Notch pathway in podocytes plays a role in the development of glomerular disease. Nat Med (2008) 14:290–8 10.1038/nm1731 18311147

[66] Sharma S Sirin Y Susztak K The story of Notch and chronic kidney disease. Curr Opin Nephrol Hypertens (2011) 20:56–61 10.1097/MNH.0b013e3283414c88 21088575 PMC3164509

[67] McLaughlin KA Rones MS Mercola M Notch regulates cell fate in the developing pronephros. Dev Biol (2000) 227:567–80 10.1006/dbio.2000.9913 11071775

[68] Chen L Al-Awqati Q Segmental expression of Notch and Hairy genes in nephrogenesis. Am J Physiol Renal Physiol (2005) 288:F939–52 10.1152/ajprenal.00369.2004 15821257

[69] Murea M Park JK Sharma S Kato H Gruenwald A Niranjan T Expression of Notch pathway proteins correlates with albuminuria, glomerulosclerosis, and renal function. Kidney Int (2010) 78:514–22 10.1038/ki.2010.172 20531454 PMC3164583

[70] Tanaka E Asanuma K Kim E Sasaki Y Oliva Trejo JA Seki T Notch2 activation ameliorates nephrosis. Nat Commun (2014) 5:3296 10.1038/ncomms4296 24526233

[71] Border WA Noble NA Transforming growth factor beta in tissue fibrosis. N Engl J Med (1994) 331:1286–92 10.1056/NEJM199411103311907 7935686

[72] Bitzer M Sterzel RB Bottinger EP Transforming growth factor-beta in renal disease. Kidney Blood Press Res (1998) 21:1–12 10.1159/000025837 9661131

[73] Kopp JB Factor VM Mozes M Nagy P Sanderson N Bottinger EP Transgenic mice with increased plasma levels of TGF-beta 1 develop progressive renal disease. Lab Invest (1996) 74:991–1003 8667617

[74] Abe Y Sakairi T Beeson C Kopp JB TGF-beta1 stimulates mitochondrial oxidative phosphorylation and generation of reactive oxygen species in cultured mouse podocytes, mediated in part by the mTOR pathway. Am J Physiol Renal Physiol (2013) 305:F1477–90 10.1152/ajprenal.00182.2013 24049142 PMC3840254

[75] Das R Xu S Quan X Nguyen TT Kong ID Chung CH Upregulation of mitochondrial Nox4 mediates TGF-beta-induced apoptosis in cultured mouse podocytes. Am J Physiol Renal Physiol (2014) 306:F155–67 10.1152/ajprenal.00438.2013 24259511

[76] Li G Li Y Liu S Shi Y Chi Y Liu G Gremlin aggravates hyperglycemia-induced podocyte injury by a TGFbeta/smad dependent signaling pathway. J Cell Biochem (2013) 114:2101–13 10.1002/jcb.24559 23553804

[77] Chen S Lee JS Iglesias-de la Cruz MC Wang A Izquierdo-Lahuerta A Gandhi NK Angiotensin II stimulates alpha3(IV) collagen production in mouse podocytes via TGF-beta and VEGF signalling: implications for diabetic glomerulopathy. Nephrol Dial Transplant (2005) 20:1320–8 10.1093/ndt/gfh837 15840669

[78] Chen S Kasama Y Lee JS Jim B Marin M Ziyadeh FN Podocyte-derived vascular endothelial growth factor mediates the stimulation of alpha3(IV) collagen production by transforming growth factor-beta1 in mouse podocytes. Diabetes (2004) 53:2939–49 10.2337/diabetes.53.11.2939 15504975

[79] Zhu Y Usui HK Sharma K Regulation of transforming growth factor beta in diabetic nephropathy: implications for treatment. Semin Nephrol (2007) 27:153–60 10.1016/j.semnephrol.2007.01.008 17418684 PMC1948024

[80] Radisky DC Epithelial-mesenchymal transition. J Cell Sci (2005) 118:4325–6 10.1242/jcs.02552 16179603

[81] Lamouille S Xu J Derynck R Molecular mechanisms of epithelial-mesenchymal transition. Nat Rev Mol Cell Biol (2014) 15:178–96 10.1038/nrm3758 24556840 PMC4240281

[82] Petermann AT Pippin J Krofft R Blonski M Griffin S Durvasula R Viable podocytes detach in experimental diabetic nephropathy: potential mechanism underlying glomerulosclerosis. Nephron Exp Nephrol (2004) 98:e114–23 10.1159/000081555 15627794

[83] Kitsiou PV Tzinia AK Stetler-Stevenson WG Michael AF Fan WW Zhou B Glucose-induced changes in integrins and matrix-related functions in cultured human glomerular epithelial cells. Am J Physiol Renal Physiol (2003) 284:F671–9 10.1152/ajprenal.00266.2002 12620921

[84] Regoli M Bendayan M Alterations in the expression of the alpha 3 beta 1 integrin in certain membrane domains of the glomerular epithelial cells (podocytes) in diabetes mellitus. Diabetologia (1997) 40:15–22 10.1007/s001250050637 9028713

[85] Chen HC Chen CA Guh JY Chang JM Shin SJ Lai YH Altering expression of alpha3beta1 integrin on podocytes of human and rats with diabetes. Life Sci (2000) 67:2345–53 10.1016/S0024-3205(00)00815-8 11065181

[86] Yamaguchi Y Iwano M Suzuki D Nakatani K Kimura K Harada K Epithelial-mesenchymal transition as a potential explanation for podocyte depletion in diabetic nephropathy. Am J Kidney Dis (2009) 54:653–64 10.1053/j.ajkd.2009.05.009 19615802

[87] Li Y Kang YS Dai C Kiss LP Wen X Liu Y Epithelial-to-mesenchymal transition is a potential pathway leading to podocyte dysfunction and proteinuria. Am J Pathol (2008) 172:299–308 10.2353/ajpath.2008.070057 18202193 PMC2312375

[88] Herman-Edelstein M Thomas MC Thallas-Bonke V Saleem M Cooper ME Kantharidis P Dedifferentiation of immortalized human podocytes in response to transforming growth factor-beta: a model for diabetic podocytopathy. Diabetes (2011) 60:1779–88 10.2337/db10-1110 21521871 PMC3114395

[89] Kagami S Border WA Ruoslahti E Noble NA Coordinated expression of beta 1 integrins and transforming growth factor-beta-induced matrix proteins in glomerulonephritis. Lab Invest (1993) 69:68–76 8331901

[90] Dessapt C Baradez MO Hayward A Dei Cas A Thomas SM Viberti G Mechanical forces and TGFbeta1 reduce podocyte adhesion through alpha3beta1 integrin downregulation. Nephrol Dial Transplant (2009) 24:2645–55 10.1093/ndt/gfp204 19420102

[91] Edge JA Dunger DB Matthews DR Gilbert JP Smith CP Increased overnight growth hormone concentrations in diabetic compared with normal adolescents. J Clin Endocrinol Metab (1990) 71:1356–62 10.1210/jcem-71-5-1356 2229292

[92] Kumar PA Brosius FC III Menon RK The glomerular podocyte as a target of growth hormone action: implications for the pathogenesis of diabetic nephropathy. Curr Diabetes Rev (2011) 7:50–5 10.2174/157339911794273900 21067510 PMC4007067

[93] Reddy GR Pushpanathan MJ Ransom RF Holzman LB Brosius FC III Diakonova M Identification of the glomerular podocyte as a target for growth hormone action. Endocrinology (2007) 148:2045–55 10.1210/en.2006-1285 17272398

[94] Kumar PA Kotlyarevska K Dejkhmaron P Reddy GR Lu C Bhojani MS Growth hormone (GH)-dependent expression of a natural antisense transcript induces zinc finger E-box-binding homeobox 2 (ZEB2) in the glomerular podocyte: a novel action of gh with implications for the pathogenesis of diabetic nephropathy. J Biol Chem (2010) 285:31148–56 10.1074/jbc.M110.132332 20682777 PMC2951188

